# A Novel Fibroblast Reporter Cell Line for *in vitro* Studies of Pulmonary Fibrosis

**DOI:** 10.3389/fphys.2020.567675

**Published:** 2020-10-09

**Authors:** Julia Nemeth, Annika Schundner, Karsten Quast, Veronika E. Winkelmann, Manfred Frick

**Affiliations:** ^1^Institute of General Physiology, Ulm University, Ulm, Germany; ^2^Boehringer Ingelheim Pharma GmbH & Co. KG, Biberach, Germany

**Keywords:** idiopathic pulmonary fibrosis, lung, myofibroblast, TGF-β, extracellular matrix, alpha smooth muscle actin

## Abstract

Idiopathic pulmonary fibrosis (IPF) is a fatal disease of the lower respiratory tract with restricted therapeutic options. Repetitive injury of the bronchoalveolar epithelium leads to activation of pulmonary fibroblasts, differentiation into myofibroblasts and excessive extracellular matrix (ECM) deposition resulting in aberrant wound repair. However, detailed molecular and cellular mechanisms underlying initiation and progression of fibrotic changes are still elusive. Here, we report the generation of a representative fibroblast reporter cell line (10-4A^*BFP*^) to study pathophysiological mechanisms of IPF in high throughput or high resolution *in vitro* live cell assays. To this end, we immortalized primary fibroblasts isolated from the distal lung of Sprague-Dawley rats. Molecular and transcriptomic characterization identified clone 10-4A as a matrix fibroblast subpopulation. Mechanical or chemical stimulation induced a reversible fibrotic state comparable to effects observed in primary isolated fibroblasts. Finally, we generated a reporter cell line (10-4A^*BFP*^) to express nuclear blue fluorescent protein (BFP) under the promotor of the myofibroblast marker alpha smooth muscle actin (*Acta2*) using CRISPR/Cas9 technology. We evaluated the suitability of 10-4A^*BFP*^ as reporter tool in plate reader assays. In summary, the 10-4A^*BFP*^ cell line provides a novel tool to study fibrotic processes *in vitro* to gain new insights into the cellular and molecular processes involved in fibrosis formation and propagation.

## Introduction

Idiopathic pulmonary fibrosis (IPF) is a progressive, irreversible and usually fatal lung disease with poor prognosis. IPF is characterized by subpleural fibrosis, subepithelial fibroblast foci, and microscopic honeycombing (Raghu et al., 2011; Wuyts et al., 2013; Lederer and Martinez, 2018; Sgalla et al., 2018). Various risk factors, including air pollution and smoking, have been associated with the development of IPF (Selman and Pardo, 2001; Kage and Borok, 2012; Liang et al., 2016; Kasper and Barth, 2017; Richeldi et al., 2017). In recent years, it has become clear that IPF is also strongly associated with genetic aberrations (Armanios et al., 2007; Seibold et al., 2011; Ryu et al., 2014; Stuart et al., 2015; Evans et al., 2016). Hence, an approach in understanding IPF pathogenesis is to consider it as a three-stage process: predisposition, initiation, and progression.

The conceptual model for the pathogenesis of IPF postulates that recurrent micro-injuries to the bronchoalveolar epithelium, superimposed on accelerated epithelial aging, result in aberrant wound repair. The reduced renewal capacity of bronchoalveolar stem cells, including alveolar type II cells, leads to reduced alveolar-epithelial cell proliferation, and secretion of profibrotic mediators (Selman and Pardo, 2001; Plantier et al., 2011; Kage and Borok, 2012; Ryu et al., 2014; Chambers and Mercer, 2015; Liang et al., 2016; Xu et al., 2016; Kasper and Barth, 2017; Richeldi et al., 2017; Lederer and Martinez, 2018). The main and most studied profibrotic cytokine is transforming growth factor beta 1 (TGF-β1). Several other cytokines play a major role in immune and inflammation responses for fibrosis formation, including interleukins (IL) like IL-13 (Zhu et al., 1999; Kolodsick et al., 2004; O’Reilly, 2013), IL-33 (Yanaba et al., 2011; Luzina et al., 2012, 2013) and IL-4 (Huaux et al., 2003; Saito et al., 2003), tumor necrosis factor alpha (TNFα) (Sime et al., 1998; Oikonomou et al., 2006; Epstein Shochet et al., 2017) as well as thymic stromal lymphopoietin (TSLP) (Datta et al., 2013; Lee et al., 2017). Profibrotic cytokines promote fibroblast activation and proliferation (Sime et al., 1997; Hinz, 2009; Luzina et al., 2015).

Fibroblast activation results in altered and increased ECM production, deposition, and accumulation (Liu et al., 2010; Bagnato and Harari, 2015). This causes remodeling processes of the pulmonary interstitium, forming scar tissue and modifying its mechanical properties (Booth et al., 2012; Parker et al., 2014). Scarring is accompanied by a strong increase in the tissue stiffness and an overall thickening of the alveolar septae (Horowitz and Thannickal, 2006; Hinz, 2009; Richeldi et al., 2017). During this progression phase, the matrix stiffness can increase from ∼ 0.5 to 15 kPa in healthy lung tissue to up to 100 kPa in fibrotic tissue depending on the measured lung compartment (Liu et al., 2010; Booth et al., 2012). The pathologically stiff matrix further propagates remodeling independent of epithelial cell dysfunction. In a feed-forward loop, increased matrix stiffness promotes additional differentiation of fibroblasts to myofibroblasts and matrix deposition (Hinz, 2009; Enomoto et al., 2013; Burgess et al., 2016; Tschumperlin et al., 2018). Overall, these processes result in the destruction of the overall alveolar architecture, leading to a strong impairment of lung function and eventually resulting in the death of the patient (Wuyts et al., 2013).

Studies regarding the onset and progress of IPF are mainly conducted in animal models by the application of fibrosis inducing agents like bleomycin (Gabazza et al., 2004; Xiao et al., 2006; Löfdahl et al., 2018). However, animal models of IPF do not fully recapitulate human pathophysiology (Lederer and Martinez, 2018). These models only partially mimic the events hallmarking IPF but it’s challenging to gain a deep insight into cellular and molecular processes. Hence, studies investigating molecular alterations of affected cell populations in response to pro-fibrotic stimuli are essential to get a more in-depth understanding of key processes responsible for development and progression of IPF. In this context, *in vitro* studies mimicking the *in vivo* situation hold great promise to elucidate molecular mechanisms underlying IPF initiation and progression. Sophisticated “lung on a chip” approaches recapitulating the alveolar microenvironment were developed and optimized by different groups (Huh, 2015; Stucki et al., 2018; Felder et al., 2019). These enable co-culture of differentiated alveolar epithelial and mesenchymal cells at air-liquid conditions whilst mimicking breathing motion and blood flow. However, the impact of these *in vitro* models depends on the use of cells representative of the *in vivo* situation.

In order to promote *in vitro* models for studying fibrotic processes, we generated an immortalized pulmonary fibroblast reporter cell line (10-4A^*BFP*^) using CRISPR/Cas9 gene-editing. 10-4A^*BFP*^ cells express nuclear blue fluorescent protein (BFP) under the promotor of the myofibroblast marker alpha smooth muscle actin (*Acta2*). To this end, we isolated primary cells from the distal lung of Sprague-Dawley rats and immortalized them using a recently described technology (Kuehn et al., 2016). We characterized several clones and validated selected clones for suitability in fibrosis studies, directly comparing responsiveness to either mechanical or chemical stimuli to responses observed in primary isolated fibroblasts. We identified clone 10-4A as a matrix fibroblast subpopulation that can be (reversibly) induced to a fibrotic state comparable to primary isolated fibroblasts. The 10-4A clone was then used for generation of a reporter cell line (10-4A^*BFP*^) expressing nuclear BFP under the promotor of the myofibroblast marker alpha smooth muscle actin (*Acta2*) using CRISPR/Cas9 technology. Finally, we evaluated the use of 10-4A^*BFP*^ cells as screening tool in plate reader assays. In summary, the 10-4A^*BFP*^ cell line provides a novel tool to study fibrotic processes in an *in vitro* co-culture system at high resolution and/or high throughput and thereby enables new insights into the cellular and molecular processes involved in fibrosis formation and propagation.

## Materials and Methods

### Chemicals and Antibodies

Human TGF-β1 was obtained from Proteintech (cat. # HZ-1011, Manchester, United Kingdom), rat IL-13 (cat. # 1945-RL-025) and rat TNF-α (cat. # 510 RT) from R&D Systems (Minneapolis, MN, United States), rat IL-33 (cat. # ab200250) from Abcam (Cambridge, United Kingdom) and rat IL-1β (cat. # 80023-RNAE) from Sino Biological (Vienna, Austria). All other chemicals were obtained from Sigma-Aldrich GmbH (Steinheim, Germany) if not stated otherwise. The following primary and secondary antibodies were used for immunofluorescence staining: αSMA (1:200, cat. # ab5694; Abcam; RRID:AB_2223021), vimentin (1:500, cat. # ab73159; Abcam; RRID:AB_1271458), EpCAM (1:200, cat. # ab71916; Abcam, RRID:AB_1603782), ABCa3 (1:500, cat. # ab24751; Abcam, RRID:AB_448287), Aqp5 (1:200, cat. # ab92320; Abcam, RRID:AB_2049171), caveolin 1 (1:200, cat. # ab2910, Abcam, RRID:AB_303405), CD45 (1:500, cat. # 12-0461-80, Thermo Fisher Scientific, Bonn, Germany, RRID:AB_2572560).

Alexa Fluor^®^ 488 goat anti-chicken (1:300, cat. # A11039; Thermo Fisher Scientific, RRID:AB_142924); Alexa Fluor^®^ 568 goat anti-rabbit (1:300, cat. # A11011; Thermo Fisher Scientific, RRID:AB_143157) Alexa Fluor^®^ 488 goat anti-mouse (1:300, cat. # A11029; Thermo Fisher Scientific, RRID:AB_138404).

### Cell Isolation and Cultivation

All lung cells were isolated from 12 to 14-week-old male Sprague-Dawley rats.

Primary alveolar type II (ATII) cells were isolated according to a modified protocol described by Jansing et al. (2018) In short, rats were anesthetized with ketamine (10%) and xylazil (2%) and injected with heparin (400 IU/kg). Lungs were perfused, removed, washed with BSS-A supplemented with EGTA, BSS-A w/o EGTA and BSS-B solution. The tissue was incubated with 0.5 mg/ml elastase (Elastin Products Co., Owensville, MO, United States) for 20 min. Then, 2 mg/ml DNase were added and the tissue was minced with sharp scissors into bits of about 1 mm^3^. The enzymatic reaction was stopped by adding FCS (GIBCO^®^ life technologies, Carlsbad, CA, United States) (37°C, 2 min). The digested tissue was filtered through gauze and nylon meshes (mesh sizes: 100, 40, and 10 μm) and the cell filtrate was centrifuged for 8 min at 130 rcf. For further cell separation, density gradient centrifugation was applied by mixing the cells in OptiPrep^TM^ Density Gradient medium (1.077 g/mL) diluted in BSS-B. The cells were centrifuged for 20 min at 200 rcf. The layer containing ATII cells was collected and supplemented with BSS-B to a total volume of 40 ml. Cells were centrifuged at 130 rcf for 8 min, resuspended in MucilAir^TM^ cell culture medium and 1 × 10^6^ cells/cm^2^ were seeded apically on 0.4 μm transparent Transwell^®^ filter inserts (Sarstedt, Nümbrecht, Germany). Purity of the ATII cells (>90%) was determined by staining with 0.4 μM Lyso Tracker Red DND 99 for 10 min (Thermo Fisher Scientific, Waltham, MA, United States) and more specifically with an ABCa3 staining. The amount of LTR positive cells was determined using a Countess II FL Automated Cell Counter (Thermo Fisher Scientific, Waltham, MA, United States).

Primary lung fibroblasts and distal lung cells were isolated according to the method of Dobbs et al. (1986) with minor modifications as previously described (Miklavc et al., 2010).

For primary isolated fibroblasts further modifications were applied:

Anesthesia, lung perfusion and removal followed the protocol described for isolation of AT2 cells. The tissue was then incubated with 0.5 mg/ml elastase (Elastin Products Co., Owensville, MO, United States) and 0.05 mg/ml trypsin at 37°C for 30 min. 2 mg/ml DNase were added, the enzymatic reaction was stopped by FCS and the digested tissue was filtered through gauze and nylon meshes (mesh sizes: 100, 70, and 40 μm). For purification of primary fibroblasts, cell suspensions were depleted of leukocytes using anti-CD45 MicroBeads (Miltenyi Biotec, Bergisch Gladbach, Germany) before fibroblasts were isolated using anti- CD90.1 MicroBeads (Miltenyi Biotec, Bergisch Gladbach, Germany) according to manufacturer’s instructions. Cells were seeded on polydimethylsiloxane (PDMS) gels or plastic substrate in MucilAir^TM^ culture medium containing 25.6 μg/ml Gentamicin ± 5 ng/mL TGF-β1 at a density of 1 to 5 × 10^5^ cells/cm^2^. Cells were cultured at 37°C, 5% CO_2_ and 95% humidity for up to 14 days. Culture media were changed every 2 days, with TGF-β1 being added freshly to the medium in corresponding experiments.

### Generation of Immortalized Cell Lines

Immortalization was performed by InSCREENeX (Braunschweig, Germany) as previously described (Lipps et al., 2018). In short: Immortalization genes were incorporated with third generation self-inactivating lentiviral vectors. Gene expression is controlled by an internal SV 40 promoter. Integration of the transgenes was verified by PCR and subsequent gel electrophoresis.

Overall, 15 immortalized cell clones were generated, all displaying characteristics of different cell populations of the distal lung. The incorporated genes used for the immortalization process for the cell line 10-4A are TAg, ID2, ID3, Rex, Nanog, and E7.

10-4A cells were maintained in the chemically defined, standardized, cell culture medium MucilAir^TM^ (Epithelix, Genève, Switzerland) containing 25.6 μg/ml Gentamicin (Thermo Fisher Scientific) at 37°C, 5% CO_2_ and 95% humidity. Cells were detached upon reaching 80% confluence using TrypLE (Thermo Fisher Scientific), centrifuged, resuspended in cell culture medium ± 5 ng/mL TGF-β1 and seeded on PDMS gels or plastic dishes at a density of 0.5 × 10^3^ to 40 × 10^3^ cells/cm^2^. Culture media were changed every 2 days, with TGF-β1 being added freshly to the medium in corresponding experiments. Cells from passage 11 to 25 were used for all experiments.

### PDMS Gel Preparation and Coating of Cell Culture Dishes

PDMS gels were prepared with the Sylgard 527 Silicon Dielectric Gel Kit (Dow Europe GmbH, Wiesbaden, Germany) as previously described (Palchesko et al., 2012) with minor modifications. In brief, component A and B were thoroughly mixed in a 1:1 ratio and added to 24 Well Culture Plates (Sarstedt, Nümbrecht, Germany) or ibiTreat μSlide 8 well (ibidi GmbH, Gräfelfing, Germany), respectively. Culture containers were kept under vacuum for 2 h to remove potential air inclusions and then incubated at room temperature for 48 h for polymerization. Fully polymerized PDMS gels were sterilized in a UV Crosslinker (GE Healthcare Europe GmbH, Freiburg im Breisgau, Germany) for 30 min and then coated with a 0.01% w/v polydopamine solution [50 mM Tris–HCl, pH = 8.5, 0.01% (w/v) Dopamine Hydrochloride] for 1 h and a 38 μg/ml rat tail collagen I solution (Advanced BioMatrix Inc., San Diego, CA, United States), diluted in Dulbecco PBS (Biochrom, Berlin, Germany; pH 7.4) over night at 37°C, respectively.

Identical coating conditions were used for culture plastic dishes w/o PDMS to ensure comparability.

### RNA Isolation, cDNA Synthesis and qPCR

Total RNA was isolated using the my-Budget RNA Mini Kit (Bio-Budget Technologies GmbH, Krefeld, Germany) with an additional DNA removal step using the RNase free DNase Set (QIAGEN GmbH, Hilden, Germany). cDNA synthesis was performed using the SuperScript^®^ VILO^TM^ cDNA Synthesis Kit (Thermo Fisher Scientific) and cDNA was diluted in a 1:3 ratio with DEPC treated H_2_O (Carl Roth, Karlsruhe, Germany) prior qPCR.

Amplification was performed on a StepOnePlus qPCR cycle (Applied Biosystems, Foster City, CA, United States) using EvaGreen QPCR Mix II (Bio-Budget Technologies). The following QuantiTect^®^ Primer assays (QIAGEN GmbH, Hilden Germany) were used: Rn_Plin2_2_SG (QT01624329), Rn_Acta2_1_SG (QT01615901), Rn_Col1a1_ 1_SG (QT01081059), Rn_Sftpc_1_SG (QT00179368), Rn_ ABCa3_1_SG (QT01587936), Rn_Vim_1_SG (QT00178724), Rn_HOPX_1_SG (QT00182693), Rn_Cav1_1_SG (QT00 398181), and Rn_Hmbs_1_SG (QT00179123). The relative quantification of mRNA expression was performed according to the method of Pfaffl (2001).

### Illumina Library Preparation and Sequencing

The Sequencing library preparation has been done using 200 ng of total RNA input with the TruSeq RNA Sample Prep Kit v2-Set B (RS-122–2002, Illumina Inc., San Diego, CA, United States) producing a 275 bp fragment including adapters in average size. In the final step before sequencing, eight individual libraries were normalized and pooled together using the adapter indices supplied by the manufacturer. Pooled libraries have then been clustered on the cBot Instrument from Illumina using the TruSeq SR Cluster Kit v3 – cBot – HS (GD-401–3001, Illumina Inc., San Diego, CA, United States) sequencing was then performed as 50 bp, single reads and 7 bases index read on an Illumina HiSeq2000 instrument using the TruSeq SBS Kit HS- v3 (50-cycle) (FC-401–3002, Illumina Inc., San Diego, CA, United States).

### mRNA-Seq Bioinformatics Pipeline

RNA-Seq reads were aligned to the rat genome using the STAR Aligner v2.5.2a (Dobin et al., 2013) with the Ensembl 84 reference genome^1^. Sequenced read quality was checked with FastQC v0.11.2^2^ and alignment quality metrics were calculated using the RNASeQC v1.18 (Deluca et al., 2012). Following read alignment, duplication rates of the RNA-Seq samples were computed with bamUtil v1.0.11 to mark duplicate reads and the dupRadar v1.4 Bioconductor R package for assessment (Sayols et al., 2016). The gene expression profiles were quantified using Cufflinks software version 2.2.1 (Trapnell et al., 2013) to get the Reads Per Kilobase of transcript per Million mapped reads (RPKM) as well as read counts from the feature counts software package (Liao et al., 2014). The matrix of read counts and the design file were imported to R, normalization factors calculated using trimmed mean of *M*-values (TMM) and subsequently voom normalized, before subjected to downstream descriptive statistics analysis.

### Western Blot

Cells were washed twice with PBS, collected with RIPA Buffer (Sigma-Aldrich) and sonicated (Sonifier 250, Branson Ultrasonics Corporation, Danbury, CT, United States) prior loading on gels. The protein concentration was determined by Pierce BCA Protein Assay (Thermo Fisher Scientific). Protein Loading Buffer and NuPAGE reducing agent (Thermo Fisher Scientific) were added in a 1:5 and 1:10 ratio, respectively. Samples were incubated at 70°C for 10 min, separated by SDS-PAGE and blotted on a nitrocellulose membrane. Immunodetection of αSMA and HSP90 was performed using Anti-alpha smooth muscle actin (1:200, cat # ab5694; Abcam, RRID:AB_2223021) and HSP90 α/β antibodies (F-8) (1:500, cat. # sc-13119; Santa Cruz Biotechnology, Dallas, TX, United States, RRID:AB_675659) in combination with fluorescent labeled secondary antibodies [1:20,000; IRDye^®^ 800CW Donkey anti-Rabbit (cat. # 926-32213, RRID:AB_621848), IRDye^®^ 680RD Donkey anti-Mouse (cat. # 926-68072, RRID:AB_10953628)] diluted in Intercept^®^ Blocking Buffer (cat. # 927-60001) (all from LI-COR Biosciences, Lincoln, NE, United States). Primary antibodies were incubated over night at 4°C, secondary antibodies for 1 h at RT, respectively. Membranes were analyzed with the Odyssey Fc Imaging System (LI-COR Biosciences).

### Immunofluorescence

For immunofluorescence staining, cells were washed with DPBS (Biochrom, Berlin, Germany; pH 7.4) and fixed in a 4% paraformaldehyde solution (dissolved in DPBS) for 10 min followed by a 1 min incubation in ice-cold 99.8% MeOH. Cells were permeabilized in a 0.2% w/v saponin solution (dissolved in DPBS) containing 10% FBS (Thermo Fisher Scientific) and 50 mM HEPES. Subsequently, cells were stained for 1 h with primary antibodies, washed twice with DPBS and stained with secondary antibodies diluted in saponin solution for 1 h. Images were taken on an iMIC digital microscope (FEI Munich GmbH, Gräfelfing, Germany) with an Olympus UApo/340 40x/1.35 Oil Iris, Infinity/0.17 lens (Olympus Europa SE & Co. KG, Hamburg, Germany) and the corresponding software (Live Aquisition v2.6.0.14).

### Generation of the *Acta2*-BFP Reporter Cell Line

CRISPR/Cas9 dependent gene editing of 10-4A cells was performed according to the method described in Ran et al. (2013).

A 20 bp single guide RNA (sgRNA) (AAACAGGAGT ATGACGAAGC), binding at the end of the coding region of the *Acta2* gene was designed by using the R&D Benchling software^3^. The sgRNA was cloned into SpCas9(BB)-2A-GFP (Addgene plasmid ID: 48138). A donor vector was designed to allow for in-frame fusion of a T2A-BFP-NLS (BFP from Evrogen, Heidelberg, Germany) cassette at the 3′ end of the *Acta2* gene. Primers used were listed in Table 1. DNA sequences flanking the sgRNA cutting site at the 5′and 3′ end were amplified from genomic DNA isolated from 10-4A cells (isolated with DNeasy Blood ans Tissue Kit, QIAGEN GmbH), and subcloned into the targeting pGEX-6P-1 Vector (GE Healthcare, 28-9546-48) using the In-Fusion kit (Clontech, Mountain View, CA, United States). 10-4A cells were co-transfected with the sgRNA/SpCas9(BB)-2A-GFP plasmid and the pGEX-6P-1 donor plasmid in a ratio of 2:1 using Lipofectamine LTX (Ratio: LTX Reagent: PLUS^TM^ Reagent, 1:1) (Thermo Fisher Scientific). The efficiency of sgRNA/Cas9-mediated integration of the BFP was evaluated after addition of 5 ng/ml TGF-β1 via fluorescence microscopy. 72 h after transfection, isolation of clonal cells was achieved by fluorescent activated cell sorting (FACS). Cells were selected for GFP and BFP expression using the BD FACSAria^TM^ III (Becton Dickinson GmbH) with the corresponding BD FACSDiva^TM^ v6.1.3 software. Single cells were seeded in 96-well plates containing cell culture medium.

**TABLE 1:** Primers used for cloning of T2A-BFP-NLS and homology arms into donor vector pGEX-6P-1.

Primer Name	Sequence 5′ → 3′
pGEX-6P-1_fwd	tgtggaattgtgagcggataac
pGEX-6P-1_rev	cattatacgcgatgattaattg
T2A-BFP-NLS_fwd	tcggctcgtataatggagggcagaggaag
	tctgct
T2A-BFP-NLS_rev	gctcacaattccacattatacctttctcttcttt
	tttgga
*Acta2*_right_homologous_right_arm_fwd	tcggctcgtataatgccctctgtgttgggcag
*Acta2*_right_homologous_right_arm_rev	acttcctctgccctcccacatctgctgga
	aggtaga
*Acta2*_left_homologous_right_arm_fwd	caggaaacagtattcgtcacgcccccaccct
*Acta2*_left_homologous_right_arm_rev	aacttccagatccgatgtaaacacatgtata
	attgtttttacttatccggtcac
pGEX-6P-1_T2A-BFP-NLS_right_arm_rev	cattatacgcgatgattaattgtcaacag
pGEX-6P-1_T2A-BFP-NLS_right_arm_fw	gagggcagaggaagtctgctaac
pGEX-6P-1_T2A-BFP-NLS_left_arm_fw	tcggatctggaagttctgttccagg
pGEX-6P-1_T2A-BFP-NLS_left_arm_rev	gaatactgtttcctgtgtgaaattgttatccg

### Functional Testing of BFP Integration

The DNA of the cell clones was extracted as described above. The region of interest was amplified by PCR and the respective products (WT: 1000 bp, with BFP insert: 1880 bp) were verified by Sanger Sequencing (Eurofins).

Additionally, in order to verify the functionality of the *Acta2* coupled BFP reporter system, cells were seeded in plastic or 5 kPa PDMS coated ibiTreat μSlide 8 well at a density of 25000/cm^2^ in MucilAir ± 5 ng/ml TGF-β1. Pictures were taken with an iMIC Digital Microscope (FEI Munich GmbH). All images were obtained using an Olympus Objective UApo/340 40x/1.35 Oil ∞/0.17 and a 405 nm excitation filter for BFP.

### Microplate Reader Assay

Cells were seeded in PDMS coated 96-well plates (Sarstedt, Nümbrecht, Germany). Respective growth factors were added 24 h post-seeding and the BFP signal was measured at indicated time points. For fluorescence measurements, cells were trypsinized, transferred to a collagen (Advanced BioMatrix Inc.) coated black 96-well plate (Sarstedt) and let adhere for 3 h. Cells were stained with Calcein AM (Thermo Fisher Scientific) for 30 min in bath solution (in mM: 140 NaCl, 5 KCl, 1 MgCl2, 2 CaCl2, 5 glucose, and 10 HEPES; pH 7.4), washed twice with PBS w/o Ca^2+/^Mg^2+^ and maintained in 200 μl bath solution during analysis of BFP signal with the plate reader (Tecan, Salzburg, Austria). Excitation wavelength were 385 nm and 485 nm and BFP and Calcein emissions were collected at 445 nm and 535 nm, respectively. BFP and Calcein signals were background subtracted and the BFP signal was normalized to the Calcein signal to adjust for cell number.

### Statistical Analysis

GraphPad Prims7 software (GraphPad, La Jolla, CA, United States) was used for statistical analysis, curve fitting and data representation. Respective tests are given within the figure legend. Data are represented as means ± SEM unless stated otherwise. Statistical significance was determined using the non-parametric Mann–Whitney-*U* test for comparison of two independent samples at the same time point. The number of experiments (N) indicates individual animals for primary fibroblasts and cells from varying passages for immortalized fibroblasts. Data was considered significant if the *p* value was < 0.05 and is indicated with an asterisk. Statistical significance is indicated in the graphs as follows: *p*-values < 0.05: ^∗^, *p*-values < 0.01: ^∗∗^, *p*-values < 0.001: ^∗∗∗^.

## Results

### 10-4A Cells Resemble Matrix Fibroblasts but Not Myo-/Lipofibroblasts

**FIGURE 1:**
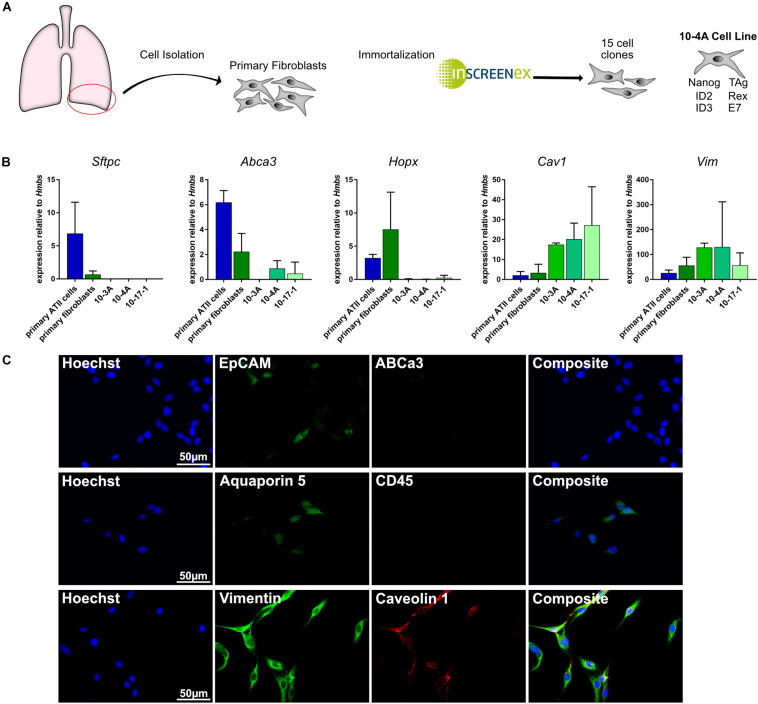
Gene and protein expression pattern of the immortalized cell lines. **(A)** Schematic representation of the immortalization process. *Right:* Genes incorporated in 10-4A cells for immortalization **(B)** Semi-quantitative RT-PCR of ATII cell (*Sftpc*, *Abca3*), ATI cell (*Hopx*, *Cav1*), and mesenchymal cell (*Vim*) marker genes. *N* = 5 **(C)** Immunofluorescent staining of 10-4A cells for expression of epithelial cell (EpCAM), alveolar type II cell (ABCa3), alveolar type I cell (aquaporin 5, caveolin 1), leukocyte (CD45), and mesenchymal cell (vimentin) marker expression. 10-4A cells predominantly express mesenchymal marker vimentin and caveolin-1, that is also expressed in lung fibroblasts. Scale bar = 50 μm.

Isolation and immortalization of primary cells from the distal lung yielded 15 individual cell clones (Figure 1A). Subsequently, cell clones were analyzed for phenotypic expression patterns resembling primary epithelial and mesenchymal cells. Expression of marker genes for alveolar type II (ATII) [*Abca3*, *Sftpc* (Beers et al., 2017)], alveolar type I (ATI) [*Hopx*, *Aqp5*, *Cav1* (McElroy and Kasper, 2004; Beers et al., 2017)], pan-epithelial [*Epcam* (Hasegawa et al., 2017)], leukocyte [*CD45* (Barletta et al., 2012)], and mesenchymal [*Vim* (Cheng et al., 2016)] cells was analyzed on the gene and protein level. Both, semi-quantitative RT-PCR and immunofluorescence data identified the presence of mesenchymal and absence of epithelial and leukocyte cell markers in several clones (Figures 1B,C). Taking into consideration that *Cav1* is also expressed in lung fibroblasts (Xiao et al., 2006) the data indicate a fibroblast phenotype of all investigated cell clones.

**FIGURE 2:**
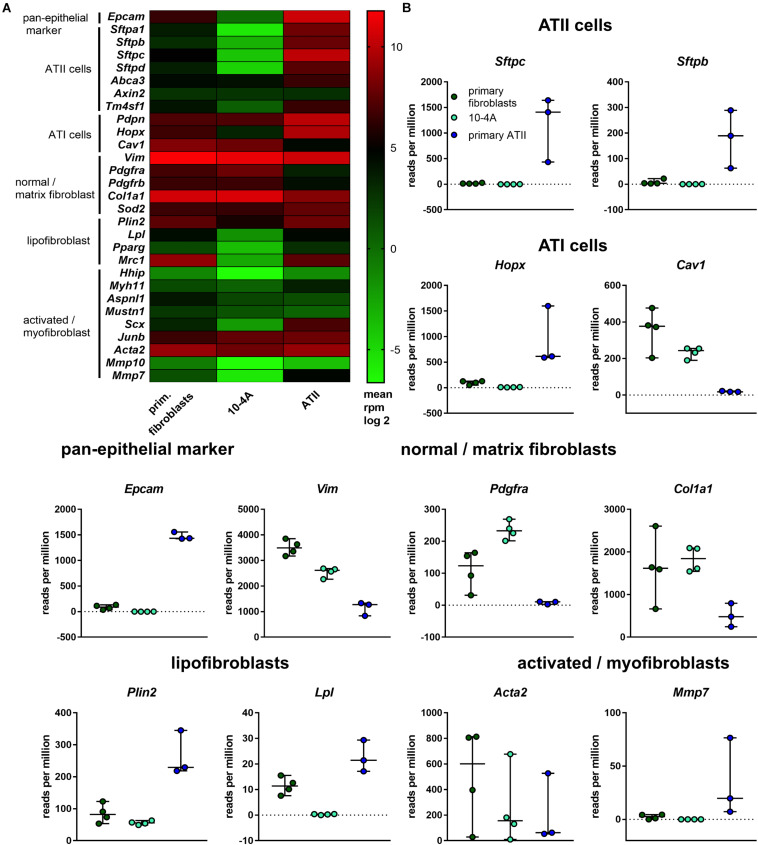
Transcriptomic profile of 10-4A compared to freshly isolated rat fibroblasts and ATII cells. **(A)** Heat map of selected pan-epithelial, ATII, ATI and different fibroblast subtype marker genes. Values are given as log2-fold change of the mean reads per million base pairs. 10-4A and primary fibroblasts, *N* = 4, primary ATII cells, *N* = 3. **(B)** Detailed presentation of rpm values obtained for selected marker genes.

Based on gene expression analysis and protein localization, clone 10-4A was selected for further analysis. In-depth characterization was performed by transcriptomic analysis. Gene expression in 10-4A cells was also compared to expression in healthy primary distal lung fibroblasts and primary ATII cells. The 10-4A cell line exhibits high expression of matrix fibroblast marker genes, in particular *Col1a1* and *Vim*, and low expression of myofibroblast marker genes. Lipofibroblast marker gene expression was low in 10-4A cells when compared to primary fibroblasts (Figure 2). Expression of specific pan- and alveolar epithelial marker genes was very low. Together these data suggest that 10-4A cells exhibit a matrix fibroblast phenotype (Zepp et al., 2017; Xie et al., 2018).

Interestingly, 10-4A exhibited high expression of *Pdgfr*α, which is associated with the capability of myofibroblast differentiation (Li et al., 2018).

### Increased Substrate Stiffness Exhibits of Myofibroblast Characteristics in 10-4A Cells

The high expression of *Pdgfr*α suggested that these cells may constitute a model to study activation/differentiation of fibroblast cells observed in pulmonary fibrosis. To test whether 10-4A cells resemble a suitable surrogate cell model for *in vitro* fibrosis studies, we first investigated the responsiveness of 10-4A cells to mechanical stimuli (Hinz, 2009). We analyzed expression of myofibroblast marker genes in response to changes in substrate stiffness. Results in 10-4A cells were compared to effects on freshly isolated primary fibroblasts.

**FIGURE 3:**
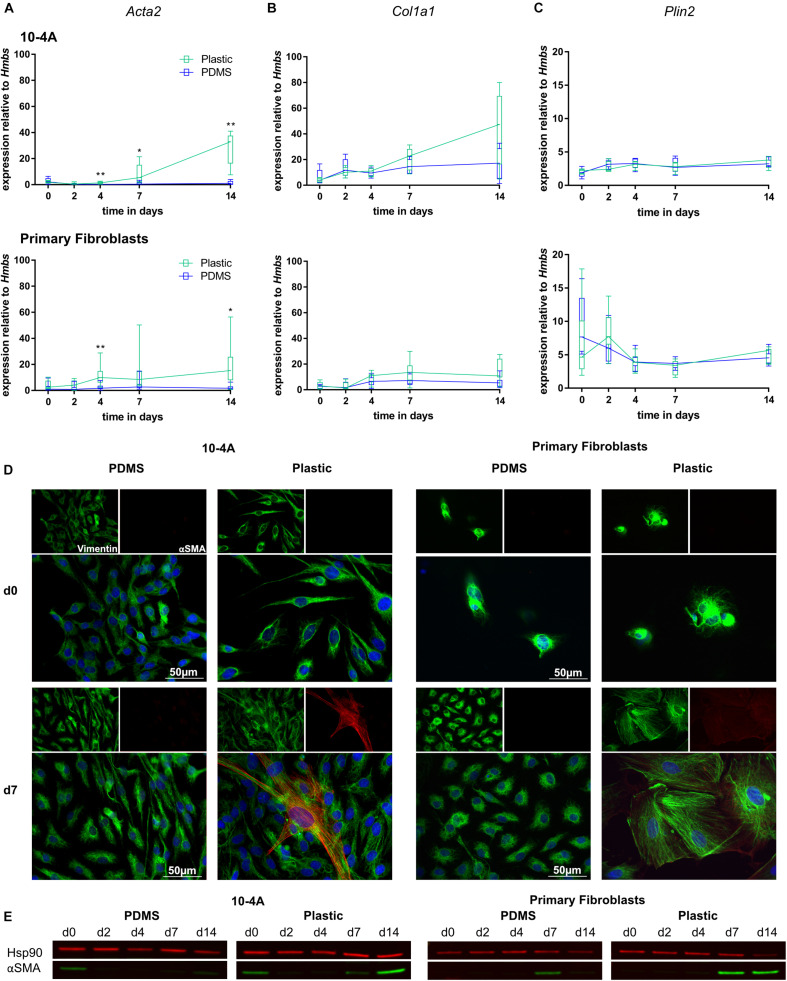
Mechanical stimulation of 10-4A cells and primary fibroblasts. Semi-quantitative RT-PCR analysis of *Acta2* **(A)**, *Col1a1* **(B)**, and *Plin2* **(C)** expression in 10-4A cells **(top graph)** and primary fibroblasts **(bottom graph)** maintained on soft PDMS or stiff plastic substrate, respectively. Data are expressed as fold expression of the housekeeping gene hydroxymethylbilane synthase (*Hmbs*). Primary fibroblast data were obtained from seven different animals, 10-4A data from five different passages. Statistical significance for the respective time points was tested with the non-parametric Mann–Whitney-*U*-Test. Statistical significance is indicated as follows: *p*-values < 0.05: *, *p*-values < 0.01: **. Box plots show data as median values, the boxes represent percentiles, the whiskers indicate the minimum/maximum. **(D)** Immunofluorescence staining of 10-4A cells **(left)** or primary fibroblasts either seeded on PDMS or plastic directly after adherence (d0) or 7 days post-seeding. Cells were stained for the mesenchymal marker vimentin (green) and the pro-fibrotic protein αSMA (red). Scale bar = 50 μm. **(E)** Western Blot for αSMA in 10-4A cells and primary fibroblasts cultured over 14 days on soft (PDMS) and stiff (Plastic) matrices, respectively. HSP90 was used as loading control.

Expression of myofibroblast marker *Acta2* and ECM component *Col1a1* were unchanged over a 14 days period in 10-4A and primary fibroblasts when maintained on soft PDMS gels with physiological stiffness (Young’s Modulus of 5 kPa) (Palchesko et al., 2012). In line, with maintenance of a quiescent phenotype, *Plin2*, a marker for lipofibroblasts, did not significantly change (Figures 3A–C). In some cases, a faint alpha smooth muscle actin (αSMA) signal (the product of *Acta2*) was detected in Western Blots at day 0 in 10-4A.

In contrast, culture on rigid plastic substrate resulted in significantly increased *Acta2* expression in 10-4A (day 4, *p* = 0.008; day 7, *p* = 0.03; day 14, *p* = 0.008) and primary fibroblasts (day 4, *p* = 0.007; day 7, *p* = 0.053; day 14, *p* = 0.03) (Figure 3A). Consistently, αSMA stress fiber formation was detected from day 7 onward, in immunofluorescence experiments and was confirmed by Western Blot (Figures 3D,E). This likely originates from αSMA expressed during cell culture in plastic culture flasks that had not been degraded by the time samples were collected (approx. 6 h after seeding on PDMs substrate). Expression of *Col1a1* and *Plin2* were not affected by stiff substrate within the 14 days culture period, suggesting that the time course might not be sufficiently long enough for full differentiation and activation of fibroblasts. A weak αSMA signal was detected in Western blots for day 0 samples from 10-4A cells.

### TGF-β1 Induces a Transient Myofibroblast Phenotype in 10-4A Cells

To further characterize the response of 10-4A cells to fibrotic stimuli, we stimulated the cells with the potent profibrotic cytokine TGF-β1.

**FIGURE 4:**
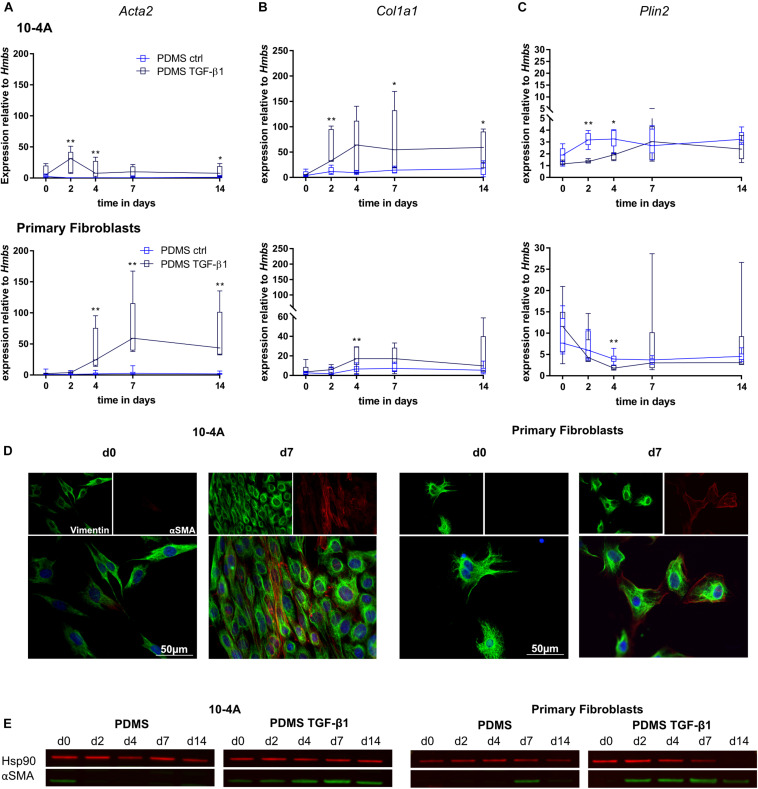
Chemical stimulation of 10-4A cells and primary fibroblasts with TGF-β1 on a physiological substrate. Semi-quantitative RT-PCR of *Acta2* **(A)**, *Col1a1* **(B)**, and *Plin2* **(C)** expression in 10-4A cells **(top graph)** and primary fibroblasts **(bottom graph)** cultured in the presence or absence of 5 ng/ml TGF-β1 on a soft PDMS substrate, respectively. Data are expressed as fold expression of housekeeping gene *Hmbs*. Primary fibroblast data were obtained from 5 different animals, 10-4A data from five independent passages. The respective time points were tested with the non-parametric Mann–Whitney-*U*-Test. Statistical significance is indicated as follows: *p*-values < 0.05: *, *p*-values < 0.01: **. Box plots show data as median values, the boxes represent percentiles, the whiskers indicate the minimum/maximum. **(D)** Immunofluorescence staining of 10-4A cells **(left)** or primary fibroblasts seeded on PDMS with administration of 5 ng/ml TGF-β1 directly after adherence (d0) or 7 days post-seeding. Cells were stained for the mesenchymal marker vimentin (green) and the pro-fibrotic protein αSMA (red). Scale bar = 50 μm. **(E)** Western Blot for αSMA in 10-4A cells and primary fibroblasts cultured over 14 days on PDMS substrate in the presence or absence of TGF-β1, respectively. HSP90 was used as loading control.

Transforming growth factor beta 1 (5 ng/ml) treatment resulted in significantly increased *Acta2* gene expression after 2 days in 10-4A cells (day 2, *p* = 0.008; day 4, *p* = 0.008; day 7, *p* = 0.057; day 14, *p* = 0.03) and after 4 days in primary fibroblasts (day 4, *p* = 0.003; day 7, *p* = 0.003; day 14, *p* = 0.003) (Figure 4A). In line, αSMA stress fiber formation was more prominent from day 2 post-seeding onward in 10-4A cells as well as primary fibroblasts (Figures 4D,E). Interestingly, the TGF-β1-induced increase in *Acta2* expression followed a transient course in the 10-4A cells, peaking at day 2 after TGF-β1 addition and returning to baseline at day 4–5. A similar trend was noticeable in primary fibroblast post day 7. In contrast to mechanical stimulation, TGF-β1 administration also resulted in significantly increased *Col1a1* (day 2, *p* = 0.008, day 7, *p* = 0.03, day 14, *p* = 0.03) and a reduced *Plin2* (day 2, *p* = 0.008, day 4, *p* = 0.02) gene expression in 10-4A cells (Figures 4B,C) and primary fibroblasts (*Col1a1* day 4, *p* = 0.003, *Plin2*, day 4, *p* = 0.003). Together these data suggest that TGF-β1 administration triggers changes observed in fibrosis in 10-4A cells and primary fibroblasts, respectively. However, the effect was not sustained over a 14-day time course, despite constant exposure to TGF-β1.

### Combination of Stiff Matrix and TGF-β1 Stimulation Leads to Strong and Persistent Expression of Myofibroblast Markers in 10-4A Cells

In pulmonary fibrosis mechanical and chemical cues act simultaneously. Therefore, we investigated the combination of mechanical and chemical stimulation seeding cells on plastic substrate and stimulated them with TGF-β1.

**FIGURE 5:**
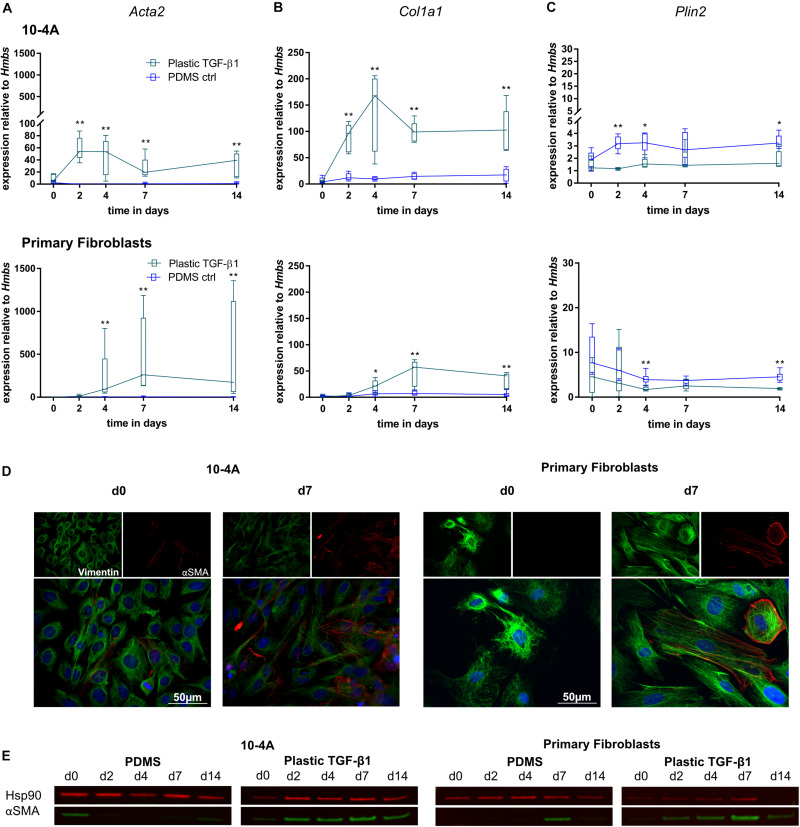
Combination of chemical and mechanical stimulation of 10-4A cells and primary fibroblasts with TGF-β1 on a physiological substrate. Semi-quantitative RT-PCR of *Acta2* **(A)**, *Col1a1* **(B)**, and *Plin2* **(C)** expression in 10-4A cells (top graph) and primary fibroblasts (bottom graph) cultured on either soft substrate (PDMS) or in the presence of 5 ng/ml TGF-β1 stiff substrate (Plastic), respectively. Data are expressed as fold expression of housekeeping gene *Hmbs*. Primary fibroblast data were obtained from five different animals, 10-4A data from five independent cell culture experiments. The respective time points were tested with the non-parametric Mann–Whitney-*U*-Test. Statistical significance is indicated as follows: *p*-values < 0.05: *, *p*-values < 0.01: **. Box plots show data as median values, the boxes represent percentiles, the whiskers indicate the minimum/maximum. **(D)** Immunofluorescence staining of 10-4A cells (left) or primary fibroblasts seeded on PDMS with administration of 5 ng/ml TGF-β1 directly after adherence (d0) or 7 days post-seeding. Cells were stained for the mesenchymal marker Vimentin (green) and the pro-fibrotic protein αSMA (red). Scale bar = 50 μm. **(E)** Western Blot for αSMA in 10-4A cells and primary fibroblasts cultured over 14 days on PDMS substrate or on Plastic in the presence of TGF-β1, respectively. HSP90 was used as loading control.

The combination of mechanical and chemical stimulation resulted in significantly increased *Acta2* gene expression in 10-4A cells from day 2 onward (day 2, *p* = 0.008, day 4, *p* = 0.008, day 7, *p* = 0.008, day 14, *p* = 0.008) and 4 days onward in primary fibroblasts (day 4, *p* = 0.003, day 7, *p* = 0.003, day 14, *p* = 0.003) (Figure 5A). This was also observed on the protein level (Figures 5D,E). Similar changes were observed for *Col1a1* (Figure 5B) (10-4A: day 2, *p* = 0.008, day 4, *p* = 0.008, day 7, *p* = 0.008, day 14, *p* = 0.008; primary fibroblasts: day 4, *p* = 0.05, day 7, *p* = 0.003, day 14, *p* = 0.003). In line, *Plin2* expression (Figure 5C) was decreased in both cell types after 4 and 2 days, respectively (10-4A: day 2, *p* = 0.008, day 4, *p* = 0.02, day 14, *p* = 0.03; primary fibroblasts: day 4, *p* = 0.005, day 14, *p* = 0.003).

Overall, the combination of mechanical and chemical stimulation induced a robust and persistent expression of myofibroblast markers in 10-4A cells and primary fibroblasts. Interestingly the transient effect observed by TGF-β1 treatment alone was counteracted by increased substrate stiffness.

### Generation of a 10-4A Reporter Cell Line to Monitor Fibroblast to Myofibroblast Differentiation in Live Cell *in vitro* Assays

**FIGURE 6:**
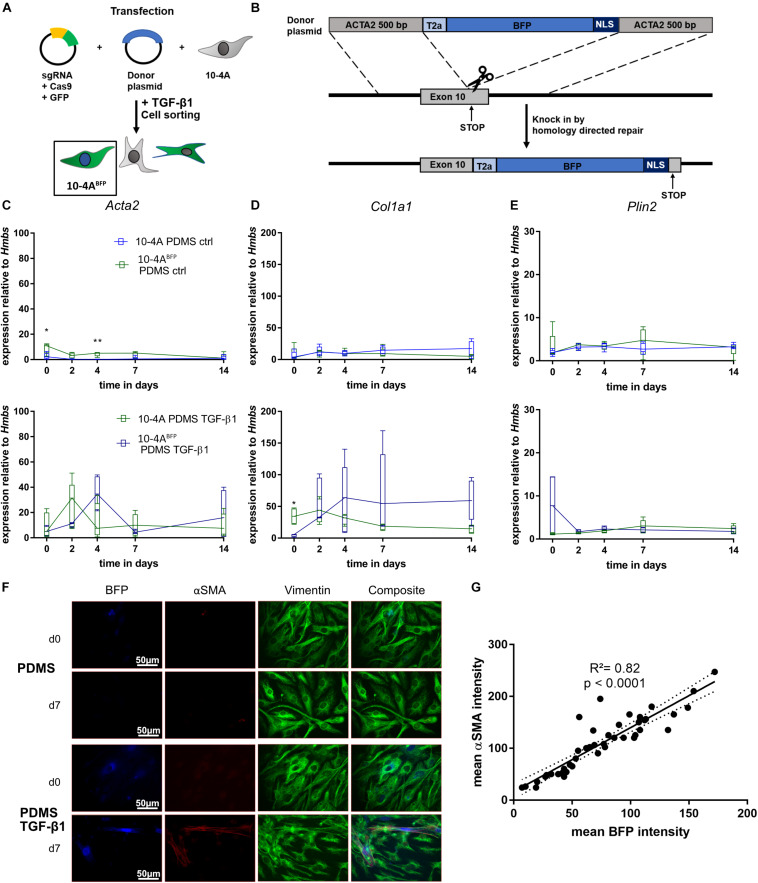
Generation and verification of an *Acta2* coupled BFP reporter cell line. **(A,B)** Schematic representation of 10-4A cell transfection. Cells were transfected with a plasmid containing Cas9 and the sgRNA binding at the desired gene (exon 10) and a donor plasmid. The donor plasmid contained a BFP flanked by a self-cleaving T2A cassette, a nuclear localization sequence and two 500 bp homologous arms of the sgRNA cutting site. After cell transfection, *Acta2* gene expression was induced by addition of 5 ng/ml TGF- β1 and cells were FACS sorted for BFP. Semi-quantitative RT-PCR analysis of *Acta2* **(C)**, *Col1a1* **(D)**, and *Plin2* **(E)** expression in 10-4A and 10-4A^*BFP*^ cells with and without administration of 5 ng/ml TGF-β1 on a soft PDMS substrate, respectively. Values are means from five individual culture experiments. The respective time points were tested with the non-parametric Mann–Whitney-*U*-Test. Statistical significance is indicated as follows: *p*-values < 0.05: *, *p*-values < 0.01: **. Box plots show data as median values, the boxes represent percentiles, the whiskers indicate the minimum/maximum. **(F)** Immunofluorescence staining of 10-4A^*BFP*^ cells seeded on PDMS with and without administration of 5 ng/ml TGF-β1 directly after adherence (d0) or 7 days post-seeding. Cells were monitored for BFP and stained for the mesenchymal marker vimentin (green) and the pro-fibrotic protein αSMA (red). Scale bar = 50 μm. **(G)** Correlation of the BFP and αSMA signal intensity within individual cells 7 days post seeding. *N* = 48 cells. A linear regression line and the corresponding indicator *R* and *p*-value show the linear dependency of BFP and αSMA signal.

In order to track fibroblast to myofibroblast differentiation within living cells, we designed a reporter system targeting the *Acta2* gene locus in 10-4A cells by generating a BFP-reporter of *Acta2* expression. We inserted a T2A-BFP-NLS sequence at the end of the *Acta2* CDS in 10-4A cells (10-4A^*BFP*^). The correct integration of the BFP sequence was verified via PCR and Sanger sequencing. Induction of myofibroblast differentiation (TGF-β1) resulted in a nuclear BFP signal in 10-4A^*BFP*^ cells (Figures 6A,B).

To exclude changes in *Acta2* gene expression arising from genetic modifications, we compared the 10-4A^*BFP*^ cell line to wildtype 10-4A cells during quiescence (on PDMS, soft matrix) and after TGF-β1 stimulation, respectively. 10-4A^*BFP*^ cells showed similar responses to PDMS and TGF-β1 stimulation as 10-4A cells when analyzing *Acta2*, *Col1a1*, and *Plin2* gene expression (Figures 6C–E), indicating no adverse effects of T2A-BFP-NLS cassette integration.

Next, we tested on the protein level, whether the BFP signal correlated with αSMA expression. TGF-β1 treatment resulted in double-positive cells, whereas cells cultured on soft substrate without TGF-β1 were negative for BFP and αSMA (Figure 6F). Correlating the αSMA and BFP signal within individual cells, confirmed a linear correlation between BFP intensity and αSMA expression (*R*^2^ = 0.82, *p* < 0.0001) (Figure 6G).

For a high throughput evaluation of fibrotic signals, a fluorescence-based 96-well assay was established. First, a linear correlation between BFP signal and cell count was verified under different culture conditions.

**FIGURE 7:**
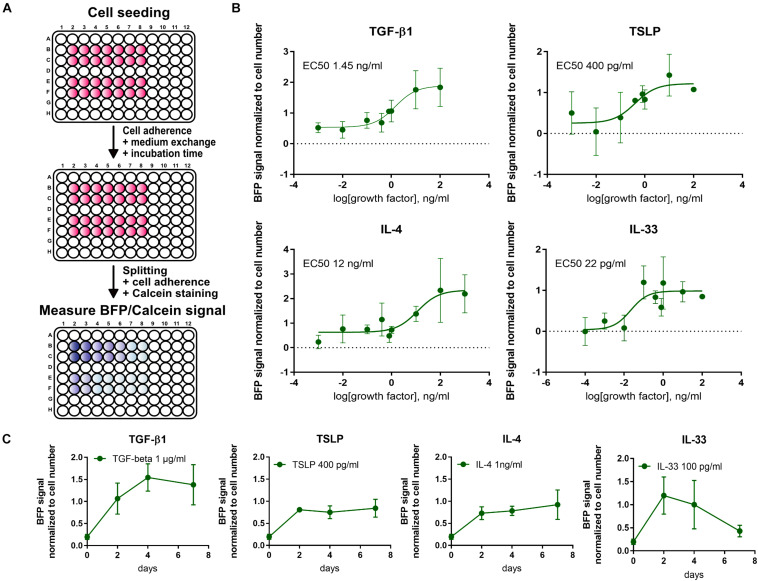
High-throughput screening using the 10-4A^*BFP*^ cell line. **(A)** Schematic of plate reader assay to study the fibrotic response of 10-4A^*BFP*^ to cytokine treatment in live cell assays. For normalization to the cell number, cells were stained with Calcein AM. **(B)** Dose-response curves of BFP expression in 10-4A^*BFP*^ cells in response to 2 days exposure with increasing doses of TGF-β1, TSLP, IL-4, and IL-33. **(C)** Time-response curve of 10-4A^*BFP*^ cells to continuous exposure to TGF-β1, TSLP, IL-4, and IL-33.

For a more thorough characterization and verification of the 10-4A^*BFP*^ cells, cytokines elevated during pulmonary fibrosis (TGF-β1, IL-33, IL-4, and TSLP) were screened to establish dose- and time-response curves (Figure 7). EC_50_ values following a 2-day incubation post-seeding were 1.45 ng/ml for TGF-β1, 400 pg/ml for TSLP, 12 ng/ml for IL-4, and 22 pg/ml for IL-33. Time course analysis of BFP signal expression revealed that the BFP signal was transiently increasing following TGF-β1 and IL-33, respectively, similar to what was observed for wildtype 10-4A cells (Figure 4). In contrast, TSLP and IL-4 show an increase in BFP expression until day 2 and afterward a stable BFP expression up to day 7.

In summary, the 10-4A^*BFP*^ cell line resembles a valuable tool for high throughput analysis of factors driving fibroblast to myofibroblast differentiation in pulmonary fibrosis, enabling screening with limited sample preparation and processing.

## Discussion

Frequently, when studying molecular and cellular mechanisms in pulmonary fibrosis, primary fibroblasts are used for *in vitro* experiments (Pierce et al., 2007; Huang et al., 2012; Pardo and Selman, 2016). However, use of primary cells is often limited by availability, restricted propagation, ethical hurdles, the cost and difficulty of repetitive cell isolations and often a heterogeneity of isolated cells within or between isolations from different donors (Kaur and Dufour, 2012). Thus, well-characterized, representative cell lines provide a useful alternative for high-throughput, live-cell assays and can be used for *in vitro* disease modeling. Genetic modifications are easily introduced to study specific signaling pathways. In addition, they offer the opportunity for long-term studies replicating diseases progression *in vitro*. The aim of our work was to generate a representative cell line to be used for *in vitro* fibrosis research. To this end, we immortalized primary rat lung cells, screened them for fibroblast characteristics and tested the induction of fibrosis after mechanical as well as chemical stimulation.

Within recent years, various cell and, in particular, fibroblast subpopulations were reported for the distal lung. Diverse roles in development, lung homeostasis, aging and injury repair were attributed to distinct populations, raising the complexity for modeling pulmonary fibrosis (Zepp et al., 2017; Xie et al., 2018). Interestingly, some myo-and lipofibroblast marker genes like *Mmp7*, *Mmp10* or *Plin2* (Adams et al., 2019; Reyfman et al., 2019; Strunz et al., 2019) were also expressed in ATII cells, emphasizing the importance to look in detail at a combination of distinct marker genes in order to assign cells to a cellular group. The presence of *Epcam* in the RNA sequencing data from primary fibroblasts likely indicates small impurities of epithelial cells, whereas the presence of *Vim* indicates minor impurities in the epithelial cell in the primary cell isolates. The presence of small fractions of other cell types after the isolation of primary lung cells is also described by other groups (Dobbs et al., 1986; Driscoll et al., 2012; Lee et al., 2018).

Regarding the fibroblast subpopulations stated by Xie et al. (2018), our 10-4A cell line neither exhibits a classical myofibroblast nor a lipofibroblast phenotype, but rather a matrix fibroblast phenotype with unexpected high expression of *Pdgfr*α. On a molecular level, the 10-4A cells could also be clearly differentiated from alveolar epithelial cells. The ATI-related genes *Cav1* as well as *Pdpn*, often used to discriminate ATI and ATII cells, are both also highly expressed in lung fibroblasts (Xiao et al., 2006; Quintanilla et al., 2019). Considering lineage-tracing and CRISPR/Cas9 *knock-in* experiments, *Pdgfr*α positive cells have been shown to give rise to either myofibroblasts or lipofibroblasts during development and to be able to differentiate into myofibroblasts in the adult lung (Ntokou et al., 2015; Zepp et al., 2017; Li et al., 2018). This directed differentiation of fibroblasts into myofibroblasts, as described for *Pdgfr*α positive cells, is one of the major requirements when studying pulmonary fibrosis. Thus, the gene and protein expression pattern of the 10-4A cell line indicates their suitability to study the mechanisms involved in the initiation and progression of pulmonary fibrosis.

Congruent with previous findings, we were also able to verify that myofibroblast differentiation can be induced mechanically (Wipff et al., 2007; Huang et al., 2012; Asano et al., 2017) and/or chemically (Chambers et al., 2003; Thannickal et al., 2003; Goffin et al., 2006; Blaauboer et al., 2011; Shi et al., 2013; El Agha et al., 2017) in 10-4A cells. Moreover, our data confirmed that fibroblasts grown on a substrate with physiological stiffness (Huang et al., 2012; Asano et al., 2017) and serum free medium (Baranyi et al., 2019) stay in a quiescent state over a time-course of 14 days. Since it is well established that fetal bovine serum exerts a heterogeneous influence on cellular behavior in cell culture due to batch-dependent variations and variable growth factor levels (Krämer et al., 2005; Mannello and Tonti, 2009; Baranyi et al., 2019), we decided to use a chemically defined, standardized, serum-free culture medium. Thereby, we were able to exclude potential variations in cellular responses due to heterogeneity of our culture conditions.

With regards to mechanical activation of the 10-4A cell line, the increase in gene and protein expression of αSMA was comparable to what has been described for primary fibroblasts as well as cell lines (Wipff et al., 2007; Huang et al., 2012; Asano et al., 2017). In line, a recent report from Tschumperlin et al., has shown that *Col1a1* mRNA levels were stable above a substrate stiffness of 0.1 kPa (Liu et al., 2010).

Transforming growth factor beta 1 in combination with a soft substrate resulted in an increase of *Acta2*, *Col1a1* and decrease of *Plin2* gene expression as well as an increase of αSMA on the protein level. These observations are in accordance to already published data in several studies of human (Chambers et al., 2003; Thannickal et al., 2003; Blaauboer et al., 2011; El Agha et al., 2017) and rodent lung fibroblasts (Goffin et al., 2006; Shi et al., 2013; El Agha et al., 2017). Interestingly, continuous stimulation with TGF-β1 induced an early but transient increase in profibrotic marker gene expression. *Acta2* expression was downregulated after 4 days in 10-4A cells continuously treated with TGF-β1. This effect was also observed in primary fibroblasts from two out of five animals after 7 days. In accordance, *Col1a1* and *Plin2* expression also adopt to control conditions for the primary fibroblasts. This indicates, that TGF-β1 stimulation alone, is not sufficient to maintain a myofibroblast phenotype and that changes in substrate stiffness are essential to induce a robust, persistent fibrotic response. The dependence of TGF-β1 on mechanical activation was already described by Shi et al. (2013). However, due to the lack of long-term studies examining the TGF-β1 effect beyond >4 days, comparable data are not available. Likewise, it has been reported, that TGF-β1 requires mechanical induction to be able to regulate myofibroblast differentiation (Hinz, 2009). This is again in accordance with our data, that the combination of chemical and mechanical stimulation is necessary to fully resemble the aspects of myofibroblast differentiation. This is also more likely to reflect the *in vivo* situation where fibrosis might be triggered by chemical stimuli but shifts to a self-perpetuating state with increasing stiffening of the lung tissue (spreading from fibrotic foci). We limited the observation time to 14 days as cellular overgrowth under fibrotic conditions, due to increased proliferation rates, resulted in inconsistent findings at observation periods beyond 14 days.

In order to accelerate and facilitate the read-out of fibroblast differentiation in a live cell, high-throughput setting, we generated an *Acta2-*BFP coupled reporter system using CRISPR/Cas9 technology. This reporter system is intended for use in live-cell, long-term screening applications. CRISPR/Cas9 can lead to off-target mutations. Therefore, we have verified correct insertion of BFP-NLS by Sanger sequencing and that 10-4A^*BFP*^ and 10-4A cells exhibit a similar behavior under physiological cell culture conditions and after fibrotic stimulation. Minor differences in expression of *Acta2* and *Col1a1* at day 0 might indicate a slightly delayed RNA turnover in 10-4A^*BFP*^ during adjustment to non-fibrotic culture conditions after transfer from culture flasks (Kitsera et al., 2007). On the protein level, αSMA signal and the respective BFP signal correlated very well, confirming appropriate reporter characteristics. Similarly, the regulation of BFP expression under the αSMA promoter could be confirmed by the targeted induction of the BFP signal after TGF-β1 administration.

Finally, in order to confirm the proper functionality and the suitability to use 10-4A^*BFP*^ cells in high throughput assays, we performed plate-reader based experiments to analyze the dose- and time-response of *Acta2* expression (i.e., myofibroblast induction) in response to exposure to distinct profibrotic cytokines. The effective concentrations were in agreement with previous reports for induction of fibrosis (Saito et al., 2003; Yanaba et al., 2011; Datta et al., 2013; Lee et al., 2017; Jones et al., 2019).

One potential limitation of the presented reporter cell line is its origin from rat rather than human. Several human pulmonary fibroblast cell lines are already available. However, in contrast to the cell line presented here, none of the currently available human cell lines has been characterized in detail with regards to the representation of a specific mesenchymal cell sub-population (Adams et al., 2019; Reyfman et al., 2019; Valenzi et al., 2019; Habermann et al., 2020; Liu et al., 2020; Travaglini et al., 2020). This might not only affect cellular responses to pro-fibrotic mechanical and chemical cues, but also affect epithelial-mesenchymal crosstalk and fibrotic remodeling when used in complex co-culture *in vitro* models (e.g., lung-on-a-chip). It is also accepted that *in vivo* animal models do not fully recapitulate all features of IPF pathogenesis (Moore et al., 2013). The reasons are not fully understood. The human lung contains structural and cellular differences to the rodent lung, however, the alveolus is one of the most conserved regions between rodents and human lungs and whether the mesenchymal and epithelial cells believed to be central to development of IPF are different is still a matter of debate. Even lipofibroblasts, a cell that’s presence has been found in rodents but has long been controversial in the human lung (Tahedl et al., 2014), has recently been identified in human lung (Liu et al., 2020; Travaglini et al., 2020). Therefore, we believe that the cell line reported here provides a valuable tool for fibrosis research, offering the opportunity to investigate molecular and cellular responses of a representative mesenchymal cell to mechanical and/or chemical stimuli with high resolution and in high throughput formats.

In summary, our data clearly demonstrate that the 10-4A cell line can be used as a valuable, novel tool for studying the onset and progression of fibrotic changes observed in pulmonary fibrosis. Additionally, the deviated 10-4A^*BFP*^ reporter cell line is a useful tool for high-throughput live cell, *in vitro* assays to directly monitor fibrotic changes over time.

## Data Availability Statement

The RNA sequencing is deposited in the Gene Expression Omnibus (accession: GSE155743).

## Ethics Statement

The animal study was reviewed and approved by the Regierungspräsidium Tübingen.

## Author Contributions

JN, AS, and MF conceived and designed the study, interpreted results of the experiments, and edited and revised the manuscript. JN, AS, and VW performed the experiments. JN, KQ, and AS analyzed the data. JN and AS prepared the figures. JN, AS, KQ, and MF drafted the manuscript. All authors approved final version of manuscript.

## Conflict of Interest

KQ was employed by the company Boehringer Ingelheim Pharma GmbH & Co. KG, Biberach, Germany. The remaining authors declare that the research was conducted in the absence of any commercial or financial relationships that could be construed as a potential conflict of interest.

## Footnotes

1 http://www.ensembl.org

2 http://www.bioinformatics.babraham.ac.uk/projects/fastqc/

3 https://www.benchling.com
